# Ion Exchange of α‑Zirconium Phosphate Prepared by Hydrothermal Synthesis

**DOI:** 10.1021/acsomega.5c11825

**Published:** 2026-03-19

**Authors:** Cecilia Hernandez, John L. Reynoso, Christian A. Santiago, Asia Sinclair, Sophia S. Bolan, Brian M. Mosby

**Affiliations:** Department of Chemistry, 8678 Rollins College, 1000 Holt Ave., Winter Park, Florida 32789, United States

## Abstract

α-zirconium phosphate (α-ZrP) is a known ion exchanger in both its amorphous and crystalline forms. The relationship between crystallinity and ion exchange has been investigated for α-ZrP prepared by reflux and direct precipitation. Hydrothermal synthesis of α-ZrP yields unique micron-sized particles with low aspect ratios, but the exchange behavior has not been thoroughly investigated. In this study, we prepare α-ZrP of varying crystallinity by hydrothermal synthesis and systematically evaluate the sodium ion exchange behavior using two distinct titration methods, incremental addition and continuous addition, at three temperatures. At room temperature, hydrothermal α-ZrP (HT ZrP) does not achieve full exchange by either titration method, but the ion uptake is improved as the exchange of the second proton is made more favorable, either by decreasing crystallinity or increasing the reaction temperature. Temperature plays a more significant role with crystalline HT ZrP and titrations by continuous addition, where exchange capacities above 95% were achieved at elevated temperatures. These insights provide a foundation for the rational design of materials with distinct ion exchange behavior and the optimization of exchange-based processes. We demonstrate the latter by the intercalation of Rhodamine 6G within zirconium phosphate at 50 °C, where the intercalation product is obtained in minutes rather than days, corresponding to an approximately 480-fold reduction in reaction time.

## Graphical abstract

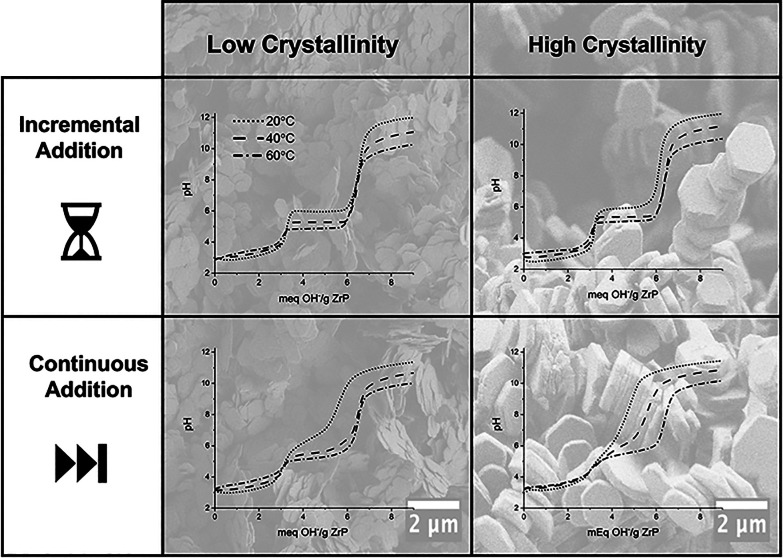

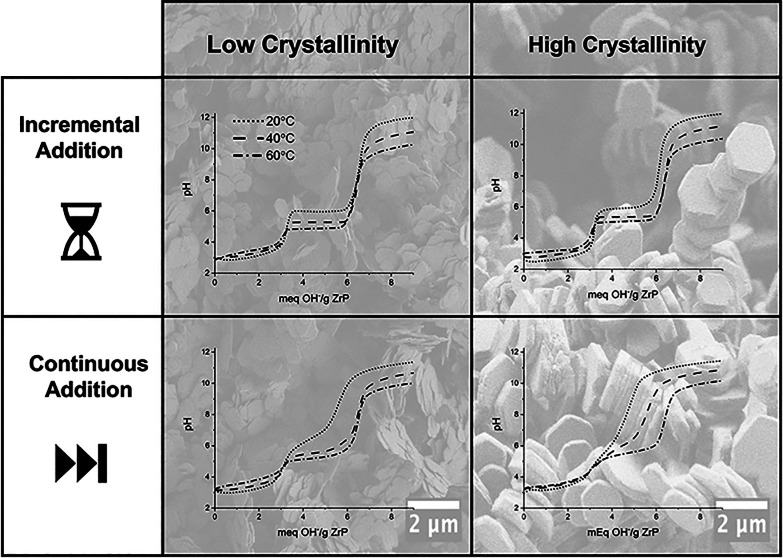

## Introduction

Ion exchange refers to the interchange of ions between two phases, typically a solid and liquid.^1^ In most cases the ions in solution enter an insoluble solid material and are retained there as they displace ions of the same charge, which subsequently enter the solution, resulting in an exchange of ions between the two phases. Although the formal scientific study of ion exchange began with Thompson and Way’s experiments with manure and soil in the 19th century, ancient accounts attributed to Aristotle and Moses describe practices consistent with ion exchange using mineral media and plant materials for water remediation.^2–5^ To date ion exchange continues to be one of the predominant approaches for remediation of soil and water, and sophisticated ion exchange materials are expected to contribute significantly to current global challenges such as the recovery of critical metals.^6–10^ While ion exchange resins account for many usages, inorganic ion exchangers possess improved chemical and thermal stability, and provide mechanistic insights based on structure–property relationships.^11^

One such inorganic ion exchange material is α-zirconium phosphate, Zr­(HPO_4_)_2_·H_2_O (α-ZrP). Amorphous zirconium phosphate was known to be an ion-exchanger, but the crystalline form, first reported by Clearfield and Stynes in 1964, allowed for the correlation of the ion exchange behavior with the structure of the material.^12,13^ The reported crystal structure is shown in Figure 1. α-ZrP consists of inorganic layers held together by electrostatic interactions and stacked along the *c*-axis. A layer is comprised of ZrO_6_ octahedra resulting from the coordination of each zirconium atom to six oxygen atoms, each from a distinct phosphate group. Each PO_4_ tetrahedron coordinates to three zirconium atoms, thereby bridging the structure, while the fourth oxygen atom is protonated and protrudes either into the interlayer region or toward the surface. The hydroxy phosphate sites are acidic and capable of undergoing reactions with cations, bases, or electroactive species, resulting in the encapsulation, or intercalation, of guest species within the layers of α-ZrP.^14^

**Figure 1:**
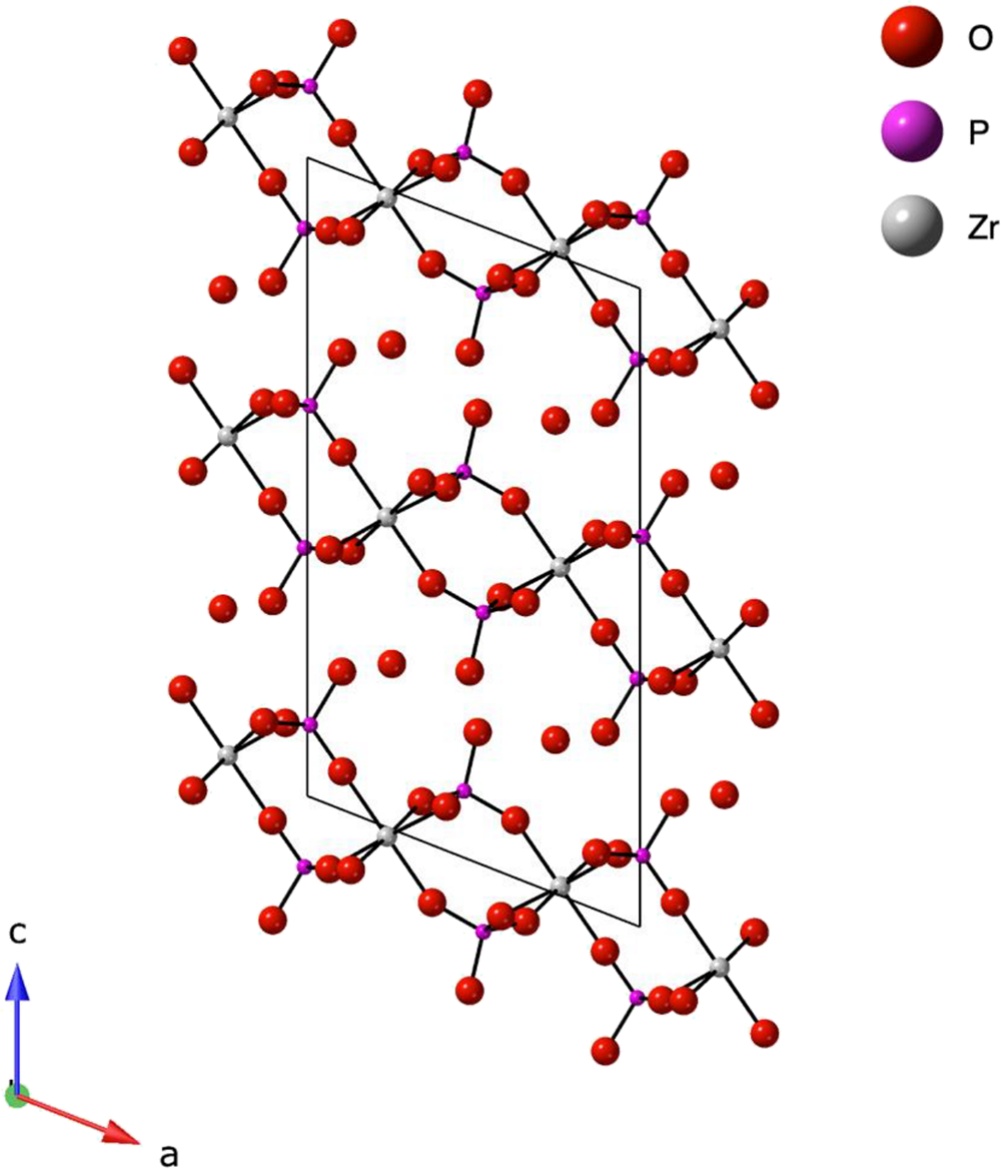
Structure of α-zirconium phosphate viewed down the *b*-axis. Hydrogen atoms of the phosphate and interlayer water molecules are omitted for clarity.

Historically, ion exchange reactions have been carried out by addition of a metal hydroxide solution to α-ZrP dispersed in a supporting electrolyte solution of the corresponding metal chloride (e.g., NaOH added to α-ZrP dispersed in NaCl). The hydroxide ions serve to neutralize the protons thereby promoting exchange throughout the material. In the case of monovalent ions, the exchange typically occurs in two steps according to eqs 1 and 2 where M^+^ represents a monovalent ion and n indicates the moles of water, which vary depending on the ion and degree of exchange.

$$\mathrm{Zr}({\mathrm{HPO}}_{4}{)}_{2}\cdot H_{2}O(s)+M^{+}(\mathrm{aq})↔\mathrm{Zr}({\mathrm{HPO}}_{4})({\mathrm{MPO}}_{4})\cdot {\mathrm{nH}}_{2}O(s)+H^{+}(\mathrm{aq})$$ 1

$$\mathrm{Zr}({\mathrm{HPO}}_{4})({\mathrm{MPO}}_{4})\cdot {\mathrm{nH}}_{2}O(s)+M^{+}(\mathrm{aq})↔\mathrm{Zr}({\mathrm{MPO}}_{4}{)}_{2}\cdot {\mathrm{nH}}_{2}O(s)+H^{+}(\mathrm{aq})$$ 2

For ion exchange to occur the incoming ions must be of sufficient size to enter the interlayer region and be stabilized within the zeolitic cavity of α-ZrP. Monovalent ions larger than potassium cannot enter the interlayer directly; in such cases, they interact only with the surface hydroxy phosphate sites and require the α-ZrP layers to be expanded by another species before they can navigate the interlayer region.^15^ The ion exchange behavior then is dependent on the hydroxy phosphate sites and an interplay between the incoming ions, the structure of α-ZrP, and the energy required to expand the layers (the intercalation energy barrier).^16^

α-ZrP has gained attention as an ion-exchange material due to its versatility, which arises from the ability to tune the structure through the preparation of functionalized derivatives and the exchange behavior through alteration of particle crystallinity.^17–20^ Additionally, α-ZrP can be prepared by reflux, hydrothermal synthesis, or direct precipitation (HF method), with each synthetic method yielding α-ZrP with distinct physicochemical properties, including size, thickness, polydispersity, and crystallinity.^21^ Recent work has highlighted the relationship between turbostatic disorder within α-ZrP and synthetic variables, such as the phosphoric acid concentration and the method utilized to prepare the materials.^22^ The disruption in layer stacking concomitant with turbostatic disorder allows for more facile incorporation of guest species within the interlayer region and is most prevalent in less crystalline preparations of α-ZrP. Essentially, the activation energy associated with the diffusion of guest species from the edge of the particles to the inner core is directly related to crystallinity, with less crystalline preparations having a lower activation energy and continuous intercalation pathway, and more crystalline preparations having a higher activation energy, resulting in a stepwise intercalation process.^23^ Such observations have been made with the intercalation of monoamines within α-ZrP, where different intercalation pathways were observed when increasing the phosphoric acid concentration utilized during reflux and when comparing lowly crystalline α-ZrP prepared by reflux with highly crystalline α-ZrP prepared by hydrothermal synthesis.^16,23^ Whereas particles prepared by reflux can reach lateral dimensions in the hundreds of nanometers, hydrothermal synthesis results in micron-sized particles.^21,24^ Additionally, hydrothermal synthesis possesses a unique crystal growth mechanism which is facilitated by oriented attachment of the flat platelet surfaces of nanocrystals, resulting in thick multidomain microcrystals with extremely low aspect ratios.^24–26^ We expect the unique physicochemical properties imparted by each synthetic method—including particle size, polydispersity, thickness, crystallinity, and degree of turbostatic disorder among others—to directly impact the diffusion pathways, ion-exchange reaction, and ultimately the achievable exchange capacities. Specifically, the thickness and strong stacking of the layers present within hydrothermal α-ZrP (HT ZrP) prepared with high phosphoric acid concentrations will limit diffusion and therefore result in incomplete exchange.

Particle morphology, especially the aspect ratio of layered solids, is known to have a direct impact on the behavior of a material and, consequently, its applications.^27–30^ However, most experiments concerning the ion-exchange of α-ZrP have been conducted with materials prepared by reflux. Recent studies contain data suggesting the ion exchange behavior of HT ZrP slightly deviates from that of α-ZrP prepared by reflux, even highly crystalline samples.^22,25^ Billinge and co-workers report exchange capacities ranging from 6.1 to 6.6 mequiv/g ZrP for samples prepared by hydrothermal synthesis.^22^ In our previous investigation, a clear trend was observed that the exchange capacity of HT ZrP decreased as the acid concentration used in synthesis increased, with 3 M HT ZrP achieving full exchange and 6 M HT ZrP and 12 M HT ZrP achieving 89 and 83% (5.9 and 5.5 mequiv/g) of the theoretical exchange, respectively.^25^ Such observations motivated the present work, which systematically investigates the ion-exchange behavior of HT ZrP to elucidate how synthesis-dependent physicochemical properties and reaction conditions influence exchange behavior. We expect our findings will allow for a more thorough understanding of ion exchange in α-ZrP and eventually the rational design of α-ZrP materials with distinctive ion-exchange behavior for targeted applications.

## Experimental Section

### Chemicals

Zirconyl chloride octahydrate (99.99%) was purchased from Inframat Advanced Materials and treated with acetone to remove excess hydrochloric acid present from the crystallization. Phosphoric acid (85%), sodium hydroxide (97.0%), sodium chloride (99.0%), and Rhodamine 6G were purchased from Sigma-Aldrich and used as received, without further purification.

### Hydrothermal Synthesis of α-ZrP

α-zirconium phosphate was prepared according to the previously reported hydrothermal method.^21^ 5.00 g of zirconyl chloride octahydrate was dissolved in 5 mL of distilled water inside a 100 mL Teflon lined reaction vessel. Distilled water and concentrated phosphoric acid were then added in appropriate quantities to produce 50 mL of phosphoric acid at the desired concentration. The reaction vessels were sealed and heated at 200 °C for 24 h. Subsequently, the samples were washed with distilled water and centrifuged to remove excess phosphoric acid. The samples were then dried at 65 °C overnight and ground with mortar and pestle to yield a powder.

### Ion Exchange

A Mettler Toledo G20S compact titrator equipped with a stirrer and temperature probe was utilized for all ion exchange experiments. In a typical experiment, 30 mL of 0.1 M sodium chloride solution was added to a plastic titration vessel followed by a known mass of α-ZrP. The vessel was sonicated until the solid was dispersed and subsequently attached to the compact titrator, where it was stirred for 1 min prior to the addition of the titrant, 0.1 M sodium hydroxide. Titrant was added at a constant rate of 1 mL/min for continuous addition experiments. For the incremental addition protocol, a set volume of titrant was added, and the solution was subsequently stirred for 3 min. A 1:1 volume (μL) to mass (mg) ratio of titrant to solid was used to determine the volume of each addition (e.g., 50 μL aliquots for 50 mg of solid). Each titration experiment was terminated after the total volume of titrant added slightly exceeded the theoretical exchange capacity of α-ZrP. Experiments above room temperature were carried out using a glass (jacketed reactor) thermostable titration vessel connected to a temperature-controlled circulating water bath. The circulating bath was set to the target temperature, allowing heated water to flow through the exterior of thermostable titration vessel. Upon the bath reaching the target temperature, the sodium chloride solution containing dispersed α-ZrP was poured into the thermostable titration vessel and stirred for 5 min to allow the solution to reach the target temperature. Subsequently, the titrant was added as previously described.

### Intercalation of Rhodamine 6G

To achieve the intercalation of Rhodamine 6G, θ-ZrP was utilized. θ-ZrP is a hydrated derivative of α-ZrP with an expanded interlayer distance, which enables encapsulation of large molecules. θ-ZrP was prepared by the method of Kijima by dropwise addition of 200 mL of 0.5 M zirconyl chloride octahydrate to an equivalent volume of 6 M phosphoric acid at 94 °C.^31^ Upon completion of the addition, a condenser was attached and the reaction was refluxed at 94 °C for 48 h. The samples were washed and purified by filtration with distilled water. The resulting paste was then weighed, redispersed in solution to maintain hydration, and utilized to prepare θ-ZrP solutions of known concentration. To perform intercalation reactions, θ-ZrP solutions of known concentration were sonicated, placed in an Erlenmeyer flask, and further diluted to a total volume of 30 mL by the addition of distilled water. Subsequently, a 20 mL solution containing a stoichiometric amount of Rhodamine 6G (0.75 mol per mole Zr) was added and the mixture was stirred for 5 days. Room temperature reactions were carried out on a standard magnetic stirrer. Intercalation reactions at elevated temperature were conducted in flasks immersed in a heated oil bath on a hot plate. To track the progress of the reaction overtime, 7 mL aliquots were removed at designated times, the solids recovered by filtration, and the Rhodamine 6G content determined by thermal analysis.

## Characterization

All α-ZrP samples were characterized by powder X-ray diffraction (PXRD), thermogravimetric analysis (TGA), and titration. PXRD experiments were carried out using a PanAlytical Empyrean X-ray diffractometer with a Cu X-ray tube at a voltage of 45 kV and current of 40 mA. TGA was conducted with a Shimadzu TGA-50 thermogravimetric analyzer in an air environment with a flow rate of 20 mL/min. Samples were heated at a rate of 10 °C/min from room temperature to 800 °C. Electron microscopy was performed with a Zeiss ULTRA-55 field-emission scanning electron microscope operated at an accelerating voltage of 1.00 kV. Images were acquired from the secondary electron detector (SE2) signal using a magnification of 5000 times and a working distance of 3.2 mm.

## Results and Discussion

Initially, ion exchange of HT ZrP was conducted with sodium ions at room temperature. Phosphoric acid concentrations of 3, 6, and 12 M were utilized to yield HT ZrP with diverse size and crystallinity, seen in Figure 2. The characteristic diffraction pattern of α-ZrP is observed for all samples along with the expected trend that crystallinity increases with the phosphoric acid concentration (Figure 2a). While both 6 M HT ZrP and 12 M HT ZrP can be regarded as highly crystalline, the 6 M sample possesses a slightly smaller fwhm suggesting a larger crystallite size. This observation aligns well with previous reports that the crystallite size of HT ZrP increases with phosphoric acid concentration until the maximum is reached with 8 M phosphoric acid, after which crystallite size decreases.^22^ Thermogravimetric analysis in Figure S1 shows the level of hydration of each sample is decreased with increasing phosphoric acid concentration. Additionally, it appears the 12 M sample retains its interlayer water and completes its condensation at much higher temperatures, suggesting a stronger binding of the water molecule resulting from a high degree of ordering among the layers. The particle sizes observed through scanning electron microscopy (SEM) in Figure 2b–d also align with the expected trends, in that both the lateral dimensions and thickness of the particles increase with acid concentration.

**Figure 2:**
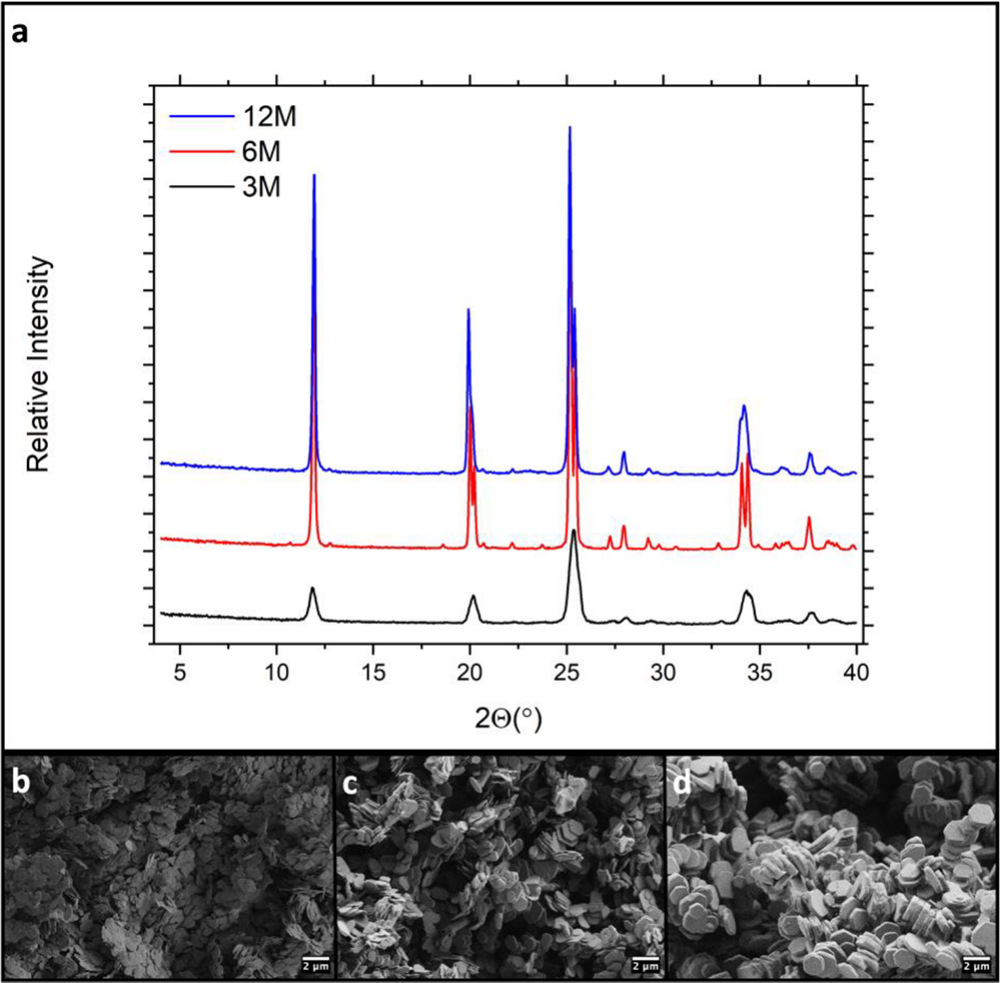
(a) PXRD patterns of HT ZrP prepared with varying phosphoric acid concentrations. (b–d) SEM images of the corresponding materials synthesized with 3, 6, and 12 M phosphoric acid, respectively.

Two methods were used to evaluate the ion exchange behavior of HT ZrP, continuous addition and incremental addition. The incremental addition method involves slow addition of titrant followed by equilibration to approximate thermodynamic equilibrium conditions and yield the intrinsic exchange capacity of each material. In contrast, the continuous addition method rapidly exposes the solid to large quantities of ions with reduced equilibration times, thereby revealing the kinetic and mass-transport limitations imposed by the synthesis-dependent physicochemical properties of HT ZrP. Comparative analysis of these methods enables discrimination between the thermodynamic and kinetic contributions to ion exchange and provides insights into the behavior of HT ZrP under dynamic reaction conditions commonly encountered in industrial and environmental ion-exchange processes.^32,33^ In each case, ion exchange experiments were carried out in triplicate and the resulting titration curves were averaged to produce a single representative curve for data presentation, as shown in Figure 3a. The first derivative of each experimental curve was then obtained, Figure 3b, allowing for the determination of the cation exchange capacity (CEC) using the inflection point corresponding to the second equivalence point of each sample (Table S1).

**Figure 3:**
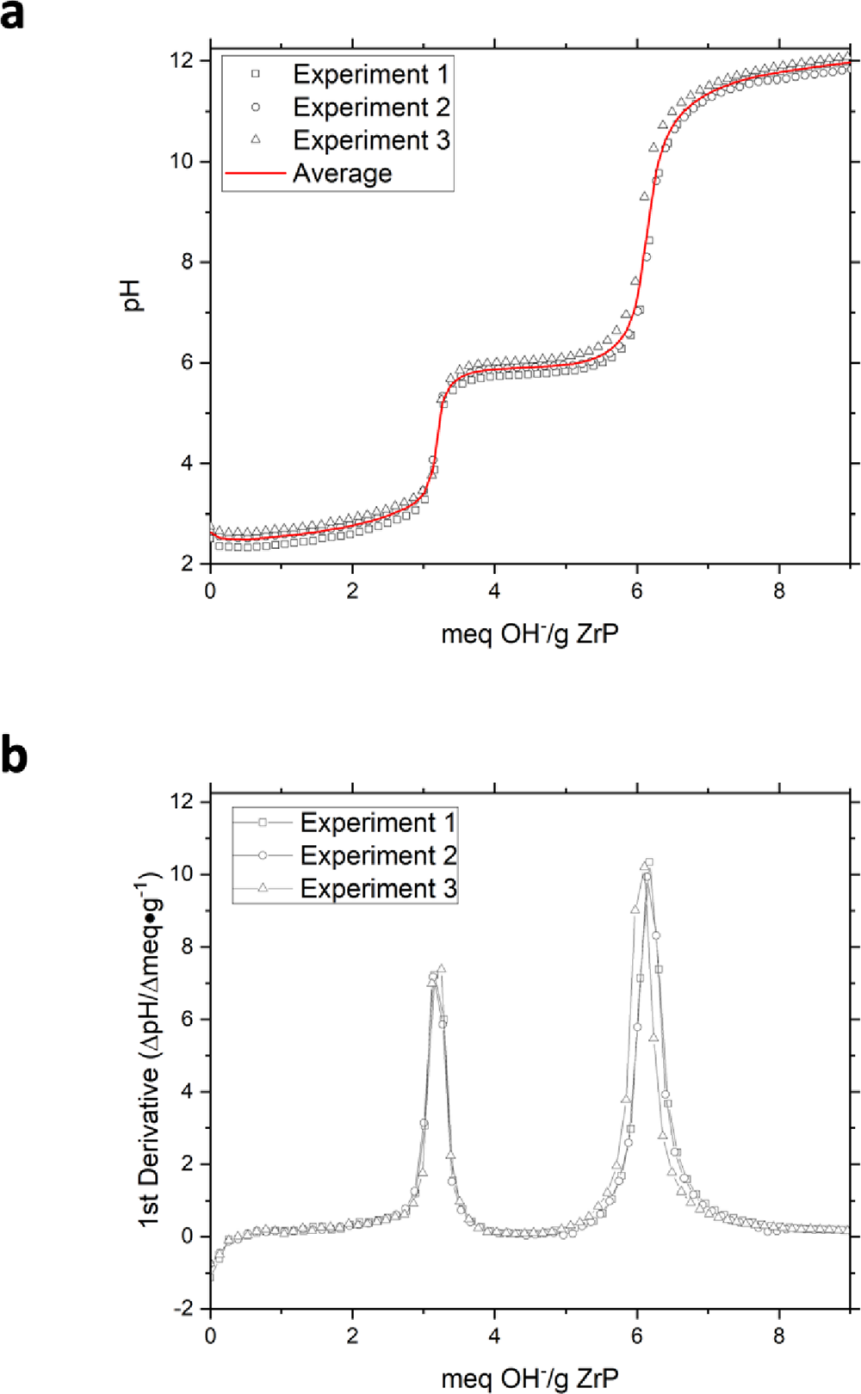
(a) Titration curves produced from triplicate data of the titration of 12 M HT ZrP with NaOH and (b) the first derivative of the experimental data.

The ion exchange curves of HT ZrP acquired using the continuous and incremental addition methods can be seen in Figure 4.

**Figure 4:**
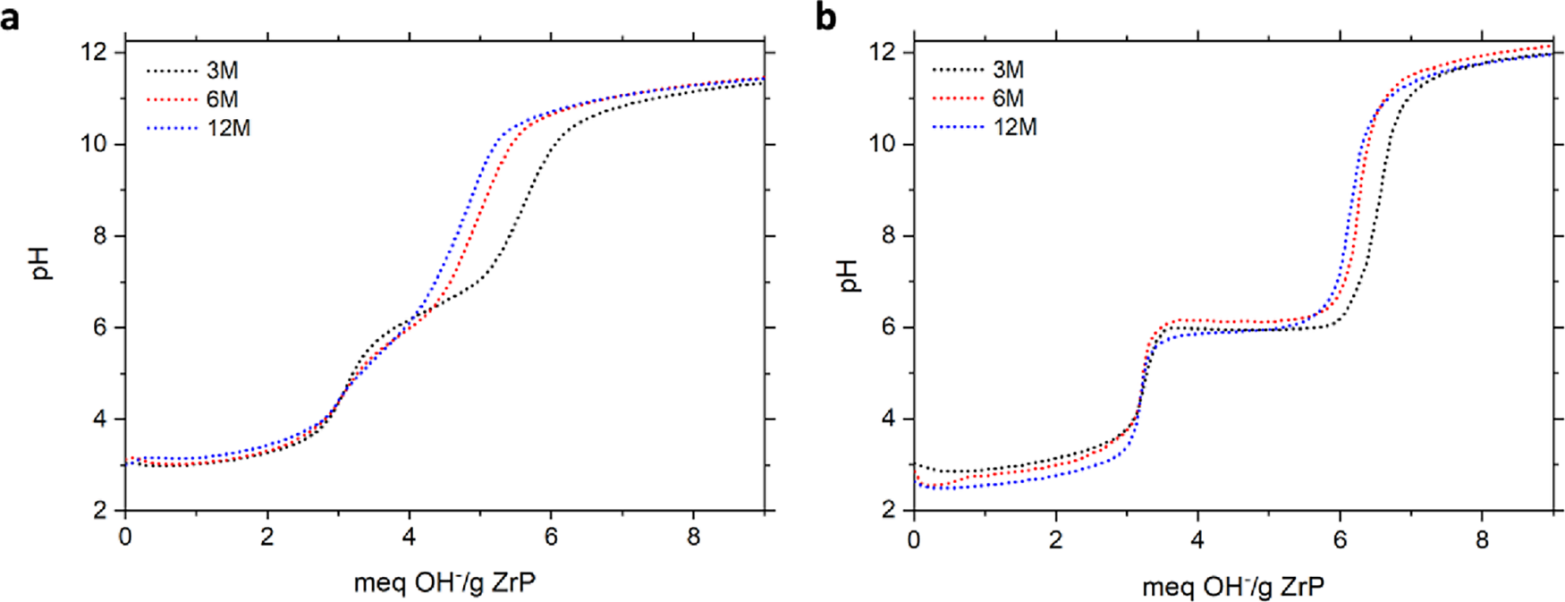
Titration curves acquired by the (a) continuous addition and (b) incremental addition of sodium hydroxide to HT ZrP prepared with 3, 6, and 12 M phosphoric acid.

Typically, a titration curve of α-ZrP exhibits a plateau where ion exchange occurs and one solid phase is converted to another, followed by an equivalence point denoting the neutralization of a proton. Each mole of α-ZrP contains two protons; therefore, the first plateau corresponds to the conversion of Zr­(HPO_4_)_2_·H_2_O to Zr­(HPO_4_)­(NaPO_4_)·5 H_2_O, and the second Zr­(HPO_4_)­(NaPO_4_)·5 H_2_O to Zr­(NaPO_4_)_2_·3 H_2_O.^34^ The theoretical exchange capacity of α-ZrP is 6.64 mequiv/g, and is achieved when the protons from all the hydroxy phosphate sites have been exchanged with sodium ions. In the case of HT ZrP, a clear trend exists such that the exchange approaches the theoretical capacity as the molarity of the phosphoric acid used in the synthesis is decreased. Essentially, an increase in crystallinity limits the efficacy of the ion exchange. The first equivalence point appears near 3.3 mequiv/g for all samples indicating there is not a strong correlation between particle crystallinity or titration method and the exchange of the first proton. This observation is consistent with the ion exchange mechanism of α-ZrP, where the first exchange involves the interaction of sodium ions with easily accessible hydroxy phosphate sites found on the edge of the particles and within disordered cavities.^35^ As a result, the first exchange is much easier as the ions can diffuse to these sites with minimal interference from the structure and at relatively low pH values.

The methods produce more divergent results when considering the exchange of the second proton. Initial examination of the curves acquired by the continuous addition method reveal the plateaus display positive slopes for all samples with the highly crystalline preparations being the steepest. In accordance with the phase rule, the positive slopes indicate more than two solid phases exist simultaneously and therefore the presence of additional solid phases of variable composition.^34^ This suggests the more crystalline preparations have more diverse composition than materials of lower crystallinity, which is the inverse of the behavior observed with reflux α-ZrP where the range of solid compositions decreased as crystallinity increased.^35^ Although the opposite trend is observed, the same rationale can be used to explain the observation. In the reflux case, the differences in the curves were primarily observed in the first exchange where disorder in the structure, which can be related directly to crystallinity, led to divergent levels of interaction among the exchanger and sodium ions. In our case, the failure of the materials to reach full exchange necessitates the presence of solids with diverse composition. We attribute this to a combination of the HT ZrP crystallinity and the rate of addition of hydroxide ions. Because the hydroxide is added continuously, there is not sufficient time for the ions to diffuse from the edges of the particles into the core where they can neutralize additional protons before more base is added. As a result, the base remains largely in solution and the addition of more base results in an increase in pH. The diffusion process is expected to require more time and energy for materials that possess a high degree of order; therefore, we expect materials with lower crystallinity to have the largest proportion of the fully exchanged solid product, which in turn will narrow the distribution of solid solution compositions and decrease the slope of the plateau. Table 1 contains the CEC of each sample, determined using the second equivalence point of each curve. The CEC is reduced as the crystallinity increases, with the 12 M sample displaying a CEC that is ∼73.6% of the theoretical capacity while the 3 M sample attains ∼86.9% of the theoretical capacity when using the continuous addition method. Both the curve shapes and the reported equivalence points agree well with the proposed explanation. Overall, the incomplete exchange is primarily a result of kinetic inhibition, where the structural order of HT ZrP creates diffusion barriers that limit access to reactive sites on the experimental time scale.

**Table 1:** Total CEC of HT ZrP Determined by Room Temperature Reaction with Sodium Ions by the Continuous Addition and Incremental Addition Methods

	cation exchange capacity (meq/g ZrP)	
	continuous addition	incremental addition
3 M HT ZrP	5.77 ± 0.05	6.60 ± 0.11
6 M HT ZrP	5.02 ± 0.10	6.27 ± 0.08
12 M HT ZrP	4.89 ± 0.08	6.14 ± 0.04

The curves produced from the incremental addition method, seen in Figure 4b, further support this hypothesis, as slowing the rate of hydroxide addition yields curves with more discernible and largely flat plateaus. Like the continuous addition method, the first equivalence point appears nearly uniform for all samples and occurs within the expected pH ranges. Additionally, the slopes of the plateaus follow the expected trend, where variation in solid solution composition is related directly to crystallinity.^35^ This suggests that the process is impacted solely by crystallinity and not the rate of addition, as in the continuous addition case. The plateaus corresponding to the conversion of the half-exchanged phase to the full exchanged phase display nearly zero slopes in all cases, indicating the pure ion exchange predominates instead of solid solution formation. The second equivalence point for the samples range from ∼92.5% to ∼99.4% of the theoretical exchange capacity, indicating a more thorough reaction of the hydroxy phosphate sites. The uptake of the materials exceeds that of the continuous addition method for all samples with the most drastic improvement observed among the samples with the highest crystallinity. It appears that the slow addition allows sufficient time for hydroxide ions to navigate the material and neutralize protons. However, full exchange is not achieved with more crystalline particles prepared by hydrothermal synthesis.

The diversity present in the second exchange appears to be a distinct feature of HT ZrP. In previous work evaluating the exchange of highly crystalline α-ZrP prepared by the reflux method, all samples were found to display identical end points, and differences in the shape of the curve were attributed to the unit cell dimensions of each material.^35^ In comparing α-ZrP prepared by direct precipitation with particles prepared by reflux, it was noted that the ion exchange process and the composition of the intermediate phases differed, however all materials were reported to achieve full exchange.^36^ The failure of HT ZrP to achieve full exchange is associated exclusively with the exchange of the second proton, which is typically attributed to the zeolitic cavities within the inner core of α-ZrP. Structurally, one zeolitic cavity forms per zirconium atom; therefore, in the half-exchanged phase, Zr­(HPO_4_)­(NaPO_4_)·5 H_2_O, there is one sodium ion per cavity. Conversion to the fully exchanged phase requires two sodium ions to reside in each cavity, which can only occur alongside dehydration and with sufficient energy to overcome electrostatic repulsions.^37^ As such, the achievement of full exchange is typically said to require overcoming an energy barrier. Therefore, the highly uniform reactive environment of more crystalline samples is expected to increase the difficulty of the exchange. Additionally, the level of order among the stacked layers is directly related to the strength of van der Waals forces holding the layers together, and therefore the activation energy associated with exchange of the second proton.^23^ Previous studies have indicated that HT ZrP displays the narrowest size distribution among all preparation methods and a unique growth mechanism that results in thicker microcrystals.^21,24^ These factors are expected to increase the strength of van der Waals forces among the layers and result in exchange sites within the particle core that are more difficult to reach compared to thinner particles, therefore the observed exchange behavior of HT ZrP seems reasonable.

We then conducted further investigations to determine whether full exchange could be achieved if sufficient energy was introduced to facilitate the exchange of the second proton. Ion exchange reactions were carried out at elevated temperatures of 40 and 60 °C using the same methods described previously. The ion exchange of 3 M HT ZrP at variable temperature can be seen in Figure 5. The exchange of the first proton is not significantly impacted by the increase in temperature. The first equivalence point occurs at largely the same position, however, the slope of the plateau increases and therefore the pH of the first exchange rises as the temperature is elevated. The first exchange process involves hydration, and it may be the case that increasing the temperature creates more disorder within the material, resulting in an increase in solid solution compositions. The opposite trend is observed for the exchange of the second proton. In this case the pH of the exchange is decreased as the temperature is elevated. The second exchange is endothermic and involves dehydration, therefore elevated temperatures increase the favorability of the reaction and result in more uniform product formation. While the plateaus from the incremental addition titration curves maintains a zero slope throughout, the slopes of the plateau obtained by continuous addition become less steep as the temperature is elevated. In all cases the elevation of temperature causes an increase in the overall exchange capacity of the material, with the most significant change being from room temperature to 40 °C. Table 2 contains the CEC of all samples as a function of crystallinity, temperature, and titration method.

**Figure 5:**
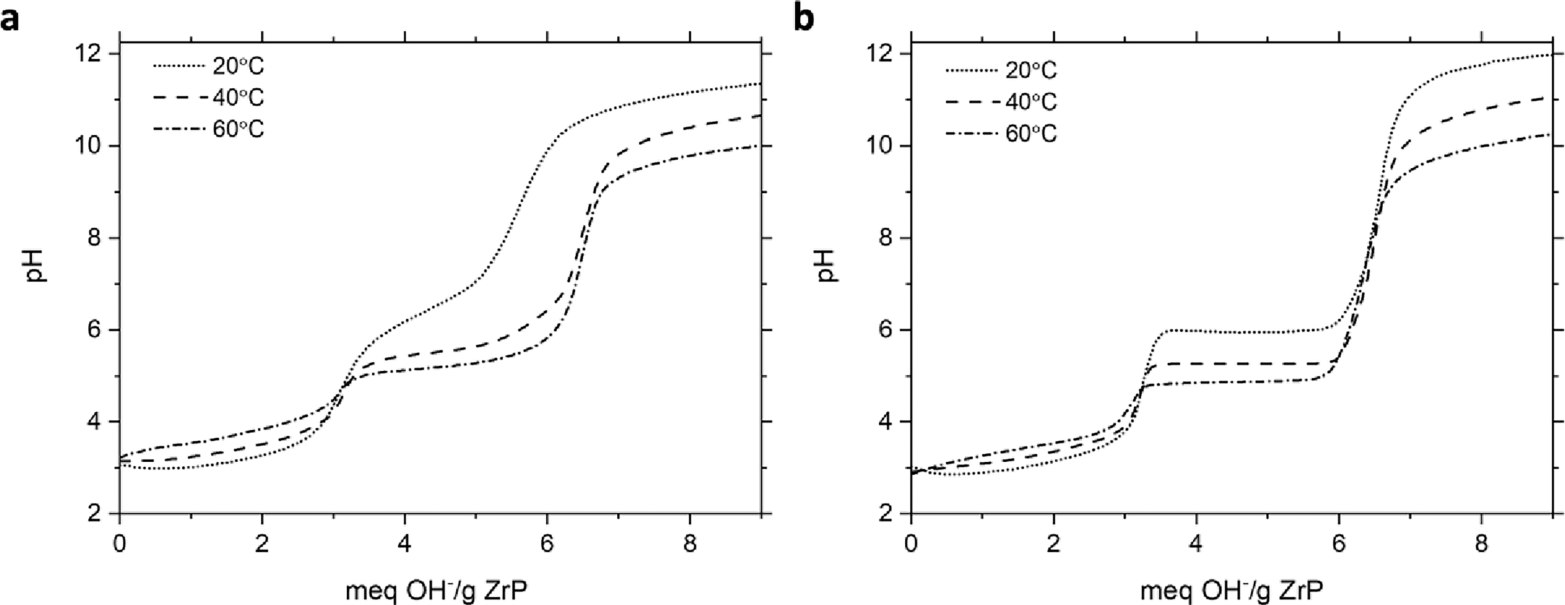
Titration of 3 M HT ZrP with sodium hydroxide by the (a) continuous addition and (b) incremental addition method at variable temperatures.

**Table 2:** Total CEC of HT ZrP Determined by Reaction with Sodium Ions at Various Temperatures by the Continuous Addition and Incremental Addition Methods

	cation exchange capacity (meq/g ZrP)					
	continuous addition			incremental addition		
	20 °C	40 °C	60 °C	20 °C	40 °C	60 °C
3 M HT ZrP	5.77 ± 0.05	6.50 ± 0.03	6.51 ± 0.02	6.60 ± 0.11	6.55 ± 0.07	6.39 ± 0.12
6 M HT ZrP	5.02 ± 0.10	5.78 ± 0.04	6.47 ± 0.11	6.27 ± 0.08	6.39 ± 0.08	6.36 ± 0.07
12 M HT ZrP	4.89 ± 0.08	5.66 ± 0.05	6.40 ± 0.02	6.14 ± 0.04	6.43 ± 0.13	6.34 ± 0.18

Relative to the theoretical exchange capacity, the ion uptake increases from 86.9% at room temperature to 98.0% at 60 °C with the continuous addition method. The increases observed using the incremental addition method are nominal and the data are not considered statistically distinct. The limited uptake observed under the continuous addition method at room temperature was attributed to the slow diffusion of hydroxide ions into the interior of crystalline α-ZrP before more hydroxide was introduced, highlighting kinetic and transport limitations imposed by the structure of ZrP. Elevating the temperature increases mobility of the ions and facilitates dehydration of sodium ions, thereby increasing the rate of ion transport and reducing the size of the hydrated ions, ultimately diminishing the influence of structural constraints on diffusion. As a result, the diversity of solid solution compositions is decreased with temperature in the continuous addition method. In the equilibrium-based exchange reaction, the decrease in pH of the second exchange indicates the process occurs more easily for the same reasons.

The results obtained with 6 M HT ZrP are seen in Figure 6. The pH of the first exchange process increases with temperature and the inverse relationship is observed for the second exchange. The slope of the plateau corresponding to the second exchange decreases with temperature, as was observed with 3 M HT ZrP. While similar trends are observed with 3 M HT ZrP, there is some distinction with the uptake behavior. In the case of 6 M HT ZrP a reaction temperature of 40 °C is insufficient to achieve full exchange using the continuous addition method. Instead, increasing the temperature produces a gradual and systematic increase of the CEC, which reaches a maximum at 60 °C. These results indicate that structural constraints on ion transport are more pronounced in 6 M HT ZrP, and higher thermal energy is necessary to overcome the diffusion and kinetic limitations associated with its more crystalline structure. The total uptake increases by 11.4% of the theoretical capacity as the temperature is elevated from 20 to 40 °C and 10.4% of the theoretical capacity as the temperature is raised from 40 to 60 °C. Overall, the CEC rises from 5.02 mequiv/g at 20 °C to 6.47 mequiv/g at 60 °C, accounting for a total increase of 21.8% of the theoretical exchange capacity, almost twice the amount observed for 3 M HT ZrP. However, when considering the equilibrium-based conditions of incremental addition, there is not a statistically significant distinction between the CEC values observed at varied temperature.

**Figure 6:**
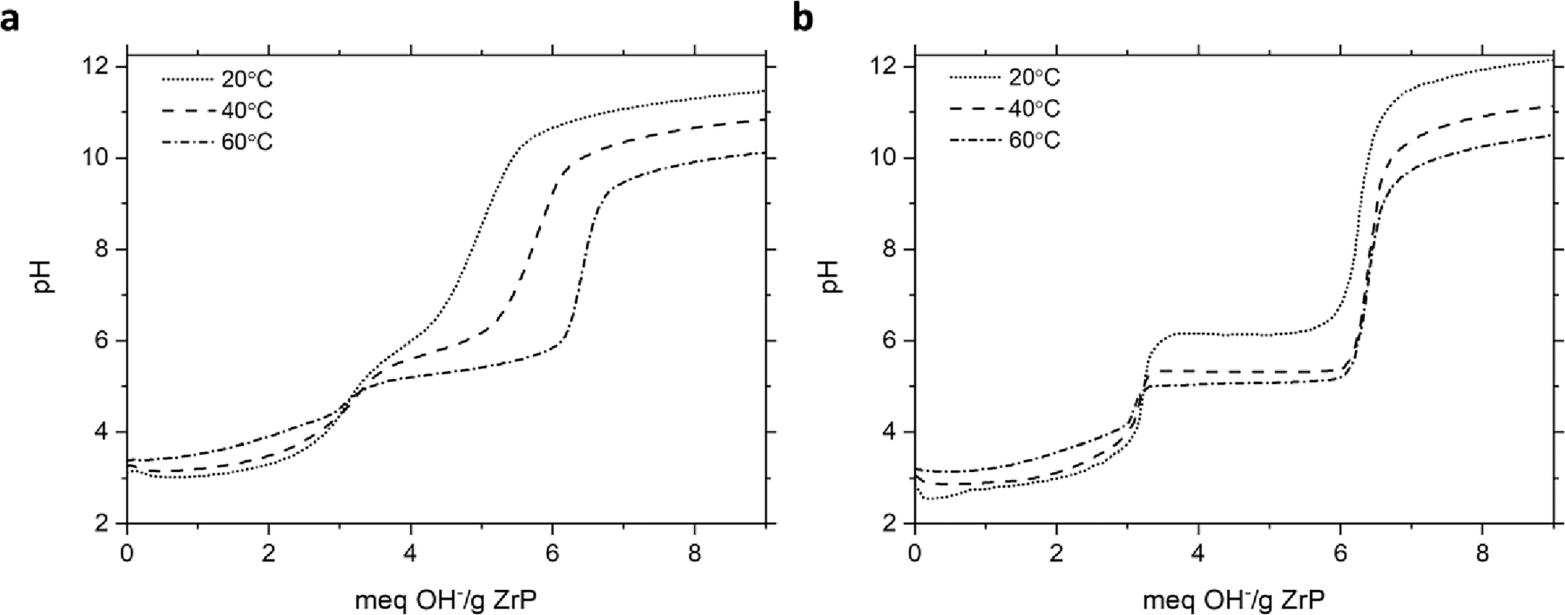
Titration of 6 M HT ZrP with sodium hydroxide by the (a) continuous addition and (b) incremental addition at variable temperatures.

Similar trends can also be observed in Figure 7 for 12 M HT samples, suggesting that once the particles obtain a certain level of crystallinity, they behave in largely comparable fashion. Overall, the data suggest the impact of elevated temperature is most significant with the continuous addition method, where increases to ion mobility and diffusion resolve the limitations observed for the room temperature exchange on the experimental time scale. While the decreases to ion hydration, faster diffusion, and shifts in equilibrium associated with higher temperatures were expected to improve the CEC of all samples, it appears the incremental addition approach leads to near equilibrium conditions in which the accessible sites are nearly saturated and therefore limited improvements to the CEC as temperature increases.

**Figure 7:**
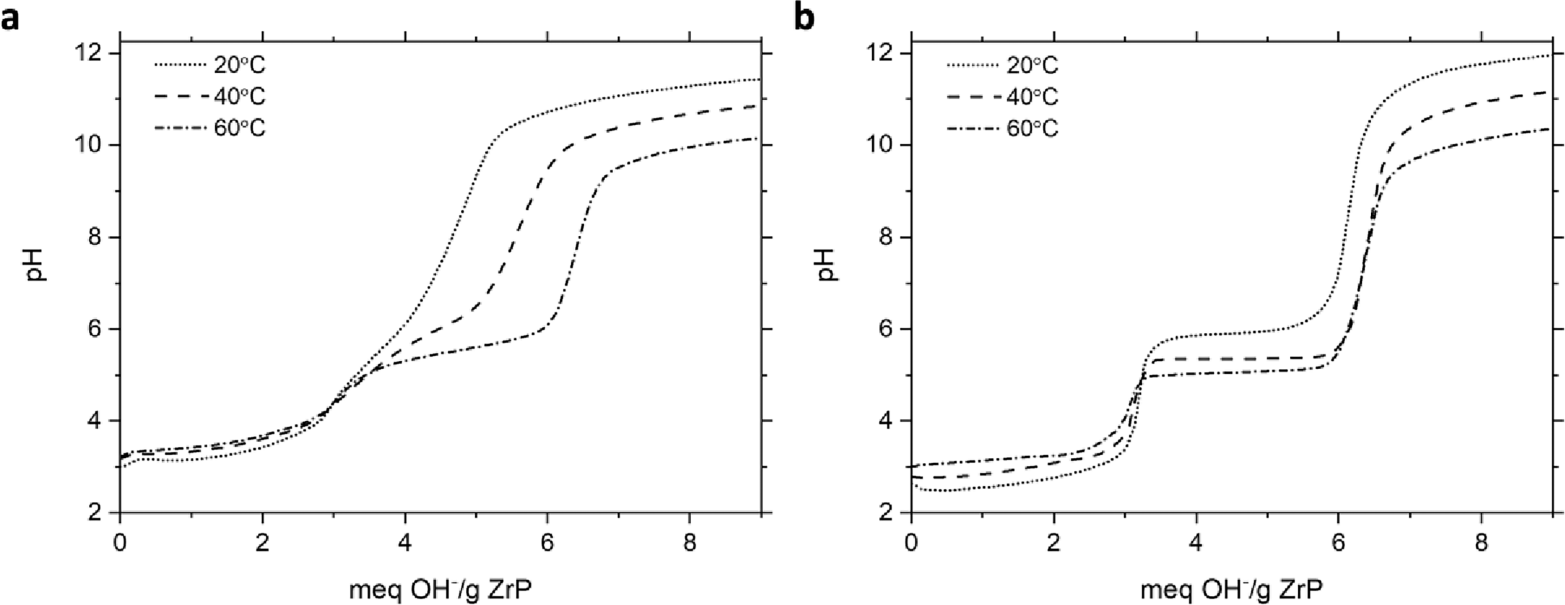
Titration of 12 M HT ZrP with sodium hydroxide by (a) continuous addition and (b) incremental addition at variable temperatures.

Investigation of the thermodynamic parameters associated with the ion exchange of HT ZrP provides additional insight into the observed exchange behavior. For all thermodynamic treatments we adopt the method of Clearfield, which treats the exchange of each proton as a reversible process. This allows for the determination of equilibrium constants using the activities of the aqueous ions involved in the exchange along plateaus of the titration curve where the solid composition is constant.^37,38^ The reactions corresponding to each ion exchange can be seen in eqs 3 and 4.

$$\mathrm{Zr}({\mathrm{HPO}}_{4}{)}_{2}\cdot H_{2}O(s)+{\mathrm{Na}}^{+}(\mathrm{aq})+4H_{2}O(l)↔\mathrm{Zr}({\mathrm{NaPO}}_{4})({\mathrm{HPO}}_{4})\cdot 5H_{2}O(s)+H^{+}(\mathrm{aq})$$ 3

$$\mathrm{Zr}({\mathrm{NaPO}}_{4})({\mathrm{HPO}}_{4})\cdot 5H_{2}O(s)+{\mathrm{Na}}^{+}(\mathrm{aq})↔\mathrm{Zr}({\mathrm{NaPO}}_{4}{)}_{2}\cdot 3H_{2}O(s)+H^{+}(\mathrm{aq})+2H_{2}O(l)$$ 4

Considering the activity of solids and water as *a* = 1, the equilibrium constant of each reaction can be determined by eq 5.

$$K=\frac{a_{H^{+}}}{a_{{\mathrm{Na}}^{+}}}$$ 5

The *K* values then allow for the direct calculation of Δ*G*. Δ*H* and Δ*S* were determined using the slope ( $\frac{-\Delta H}{R}$ ) and *y* intercept ( $\frac{\Delta S}{R}$ ) of van’t Hoff plots prepared using the *K* values from the variable temperature experiments (Figures S2–S4). The uncertainty of the enthalpy and entropy terms were determined by the linear regression analysis associated with the fitting of the data. The uncertainty of the proton and sodium ion activities dictate the uncertainty of both the equilibrium constant and free energy terms. The calculated thermodynamic parameters for the exchange of the first and second proton can be found in Tables 3 and 4, respectively, along with literature values reported for crystalline α-ZrP, prepared by the reflux of zirconyl chloride octahydrate with 12 M phosphoric acid for 330 h (ZrP 12:330).

**Table 3:** Thermodynamic Data for the Ion Exchange of the First Proton of α-ZrP with Sodium Ions at 20°C

	Δ*G* (kcal/mol)	Δ*H* (kcal/mol)	*T*Δ*S*(kcal/mol)	Δ*S* (e.u.)
3 M HT ZrP	2.51 ± 0.03	–3.27 ± 5.8 × 10^–16^	–5.89 ± 5.5 × 10^–16^	–19.75 ± 1.9 × 10^–15^
6 M HT ZrP	2.27 ± 0.03	–3.26 ± 0.04	–5.87 ± 0.04	–19.70 ± 0.01
12 M HT ZrP	2.03 ± 0.03	–6.45 ± 0.51	–8.63 ± 0.49	–28.96 ± 1.65
ZrP 12:330	2.33 ± 0.03^a^	–6.90 ± 0.10^a^	–9.23 ± 0.12^a^	–31.0 ± 0.1^a^

a Values from references 37,38 , in which experiments were conducted at 25 °C.

**Table 4:** Thermodynamic Data for the Ion Exchange of the Second Proton of α-ZrP with Sodium Ions at 20°C

	Δ*G* (kcal/mol)	Δ*H* (kcal/mol)	*T*Δ*S*(kcal/mol)	Δ*S* (e.u.)
3 M HT ZrP	6.66 ± 0.03	12.07 ± 1.13	5.56 ± 0.70	18.63 ± 2.35
6 M HT ZrP	6.90 ± 0.03	11.96 ± 2.46	5.27 ± 2.35	17.67 ± 7.88
12 M HT ZrP	6.57 ± 0.03	9.34 ± 0.90	2.87 ± 0.87	9.62 ± 2.90
ZrP 12:330	6.45 ± 0.03^a^	6.45 ± 0.15^a^	0.00 ± 0.15^a^	0.0 ± 0.1^a^

a Values from references 37,38 , in which experiments were conducted at 25 °C.

The free energy change associated with the exchange of the first proton is largely similar for all preparations regardless of acid concentration or synthetic method. This agrees well with the proposed exchange mechanism which relegates the first exchange to disordered and easily accessible sites. The slightly positive values also indicate the reaction is not spontaneous; in our case, hydroxide ions were added to propel the reaction through deprotonation of reactive sites and subsequent expansion of the interlayer. Both Δ*H* and Δ*S* appear to decrease with increasing acid concentration. The enthalpy change of the exchange is attributed primarily to the enthalpy changes accompanying hydration and dehydration of the ions, the heat consumed in the breaking of P–OH bonds associated with deprotonation, and the heat released from the binding of sodium ions to the phosphate sites within the cavity.^39^ We expect more crystalline preparations of α-ZrP to provide more uniform and stronger binding within the zeolitic cavity and therefore a more negative contribution to Δ*H*. Although the first exchange is accompanied by an expansion of the interlayer (7.6–11.8 Å) and an increase in hydration (monohydrate to pentahydrate), the negative Δ*S* values indicate the resulting stoichiometric hydration complex reduces the stacking disorder present among the layers. Well-ordered inorganic layers yield uniform interlayer environments where the sodium ions and water molecules are constrained in precise locations by a series of electrostatic interactions with the hydroxy phosphate sites. This results in a greater loss of entropy relative to less crystalline samples, where structural heterogeneity allows some disorder to persist.

Data for the exchange of the second proton can be seen in Table 4. As was the case for the exchange of the first proton, the Δ*G* values are similar among all ZrP preparations and both Δ*H* and Δ*S* decrease with crystallinity. However, the exchange of the second proton has higher Δ*G* and Δ*H* values relative to the first exchange, suggesting the reaction is less favorable, in agreement with previously discussed structural arguments concerning the ion exchange mechanism.

Conversion from the half to the full exchanged phase requires the loss of two moles of water accompanying the sodium ions and the inclusion of an additional sodium ion within the zeolitic cavity. The stronger binding of sodium ions to phosphate sites in more crystalline α-ZrP adds more substantial exothermic contributions and results in lower Δ*H* values. However, the values remain positive due to the high enthalpic cost of deprotonating the highly stable hydroxy phosphate sites. The Δ*S* values indicate reorganization of α-ZrP upon further exchange, with the magnitude of the entropy change strongly dependent on crystallinity. While the reduction of the interlayer more effectively confines water molecules yielding negative contributions to the entropy change, the corresponding dehydration releases water and therefore disrupts the highly stable electrostatic interactions present within the well-ordered hydration complex leading to large positive entropic contributions. More crystalline preparations are structurally constrained and possess fewer hydration microstates resulting in significantly fewer perturbations from ion-exchange and the observed trend that Δ*S* approaches zero as crystallinity increases. The rigid inorganic layers of α-ZrP along with the regular arrangement of phosphate sites capable of forming strong electrostatic interactions and extended hydrogen bonding networks, distinguish the ion exchange thermodynamics from those of other layered materials. The hydration that accompanies ion exchange within layered double hydroxides also produces a more structured material, but the decrease in configurational entropy is insignificant relative to the large increase in vibrational entropy due to weak interactions among the ions, the solvent, and the inorganic layers.^40,41^ In contrast, montmorillonite clays exhibit positive entropy changes during ion exchange resulting primarily from the hydration entropy of interlayer ions and the accompanying volume change.^42^

Overall, the thermodynamic values associated with the ion exchange of HT ZrP more closely resemble the crystalline reference as the crystallinity increases, with 12 M HT ZrP being very comparable, especially for the exchange of the first proton. Although 6 M HT ZrP and 12 M HT ZrP can both be regarded as crystalline based on PXRD and display similar ion-exchange behavior, the thermodynamic data suggests the 12 M sample behaves in a more ideal manner during the ion exchange process. The reduction in endothermic and entropic contributions to the ion exchange from 6 M HT ZrP to 12 M HT ZrP indicate well ordered, rigid layers, with more uniform exchange sites. This agrees well with our TGA data which indicates the interlayer water is bound more tightly in 12 M HT ZrP than 6 M HT ZrP (Figure S1). Considering the 12 M HT ZrP can be more than double the lateral dimensions of 6 M HT ZrP and hundreds of nanometers thicker, it seems reasonable that the structure would maintain more order and stronger binding of interlayer species.^21,24,25^ The ion exchange behavior then is not solely a function of bulk crystallinity but factors such as the local structure, interlayer hydration, and particle morphology also impact the favorability of the reaction.

Although these findings focus exclusively on HT ZrP, we expect they have implications for other phases of zirconium phosphate and layered solids. Specifically, the observation that the continuous addition method at elevated temperatures yields similar levels of uptake as the gradual equilibrium-based exchange may be applied directly to the intercalation of guest species within layered solids. Although intercalation can be achieved by mechanisms other than ion-exchange, in all cases the diffusion-based interaction of the guest species with the layers closely aligns, and the primary difference pertains to the way the guest is stabilized within the interlayer. The rate limiting step in intercalation will likewise concern the diffusion of guest species from the edges of particles and disordered sites into the core. As previously discussed, this diffusion will be more challenging for preparations that are highly ordered, possess strong van der Waals interactions, and limited flexibility such as HT ZrP. Therefore HT ZrP and other highly crystalline solids have limited applicability for intercalation, especially of large molecules with sizes that exceed the separation of the layers. To optimize intercalation reactions researchers have adopted methods to weaken the attraction between layers and make the uptake of large guest more facile. The most prevalent approach for zirconium phosphates was pioneered by Colón and co-workers who demonstrated the direct intercalation of Ru­(bpy)^2+^ into θ-ZrP, a highly hydrated form of ZrP with the α type structure and an expanded interlayer (10.4 Å), using room temperature mixing for 5 days.^43^ Similar methods were used to intercalate other large molecules such as doxorubicin, insulin, ferrocenium, and Rhodamine 6G.^44–47^ In each case a suspension of θ-ZrP is put in contact with a large quantity of the prospective guest molecule and allowed to react over a prolonged period of time. The initial conditions of intercalation then closely resemble the continuous addition titration protocol, where the zirconium phosphate is initially in contact with a large quantity of guest molecule. Based on our findings with HT ZrP, we hypothesize intercalation reactions can be achieved more efficiently using elevated temperature to facilitate diffusion of guest molecules.

Here, we perform intercalation experiments with θ-ZrP and Rhodamine 6G, a large organic dye, at room temperature and 50 °C, and use a combination of structural and thermal analysis to evaluate the impact of temperature on the progress of the reaction. Initially, PXRD was used to confirm the successful intercalation of Rhodamine 6G, Figure 8, where the resulting powder diffraction patterns are consistent with the previous report of the intercalation compound.^48^ The first peak in all the diffraction patterns corresponds to an interlayer distance of nearly 19Å and provides direct structural evidence for the expansion of the interlayer through the encapsulation of Rhodamine 6G. However, for the room temperature intercalations, a broad peak appears near 12° 2Θ from 0.25 to 1 h, which decreases in intensity with time. This peak is representative of α-ZrP and indicates Rhodamine 6G has not diffused to some regions of the material yielding an incomplete intercalation. The disappearance of this peak at 24 and 120 h indicates that at room temperature the diffusion of the guest molecule and therefore the successful intercalation require extended time. The diffraction patterns observed for the intercalation at elevated temperature are nearly identical regardless of the time, indicating intercalation proceeds rapidly and produces a highly ordered material even at times as short as 0.25 h. In fact, the diffraction patterns of the products of the intercalation reaction at room temperature for 120 h and 50 °C for 0.25 h appear to be identical.

**Figure 8:**
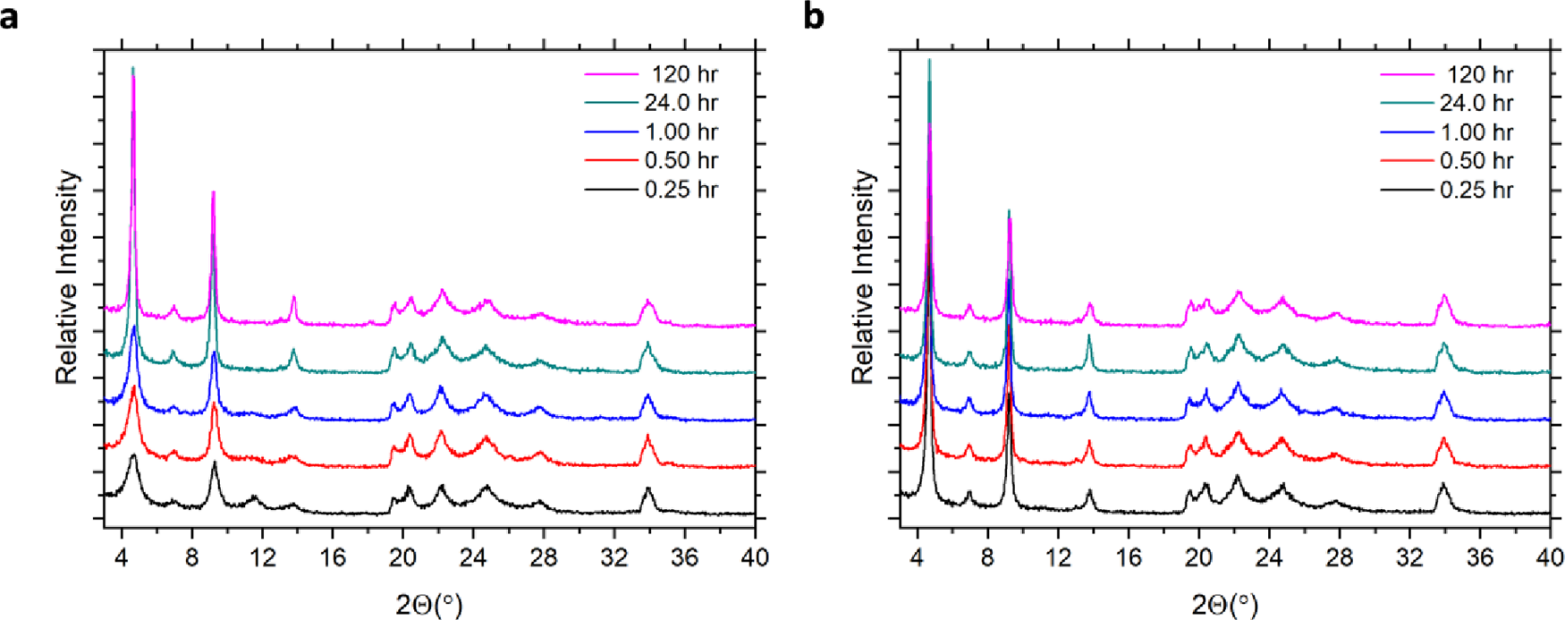
PXRD patterns of the solid products recovered from the intercalation of Rhodamine 6G with θ-ZrP over a 5-day time scale at (a) room temperature and (b) 50 °C. All data in (a) and (b) are presented on the same absolute intensity scale and with a constant vertical offset.

The corresponding thermogravimetric analysis data comparing the longest time frame at room temperature with the shortest at elevated temperature can be seen in Figure 9. The derivative of the TGA indicates there are four weight loss events associated with the thermal degradation of the Rhodamine 6G intercalation compound. The TGA of α-ZrP typically proceeds in three weight loss events, loss of surface adsorbed solvent, the interlayer water molecule, and the condensation of the phosphate to produce zirconium pyrophosphate as the product of thermal degradation.^49^ The additional weight loss event in the intercalation compound is attributed to the decomposition of the encapsulated organic molecule. The loss of surface adsorbed solvent occurs below 100 °C and dehydration of the interlayer is complete at 200 °C. The thermal degradation of Rhodamine 6G occurs in two steps. The first weight loss occurs from ∼300 °C to ∼375 °C and the second corresponding to the decomposition of the bulk of the molecule occurs between 400 and 600 °C, which also overlaps with the condensation of the phosphate.

**Figure 9:**
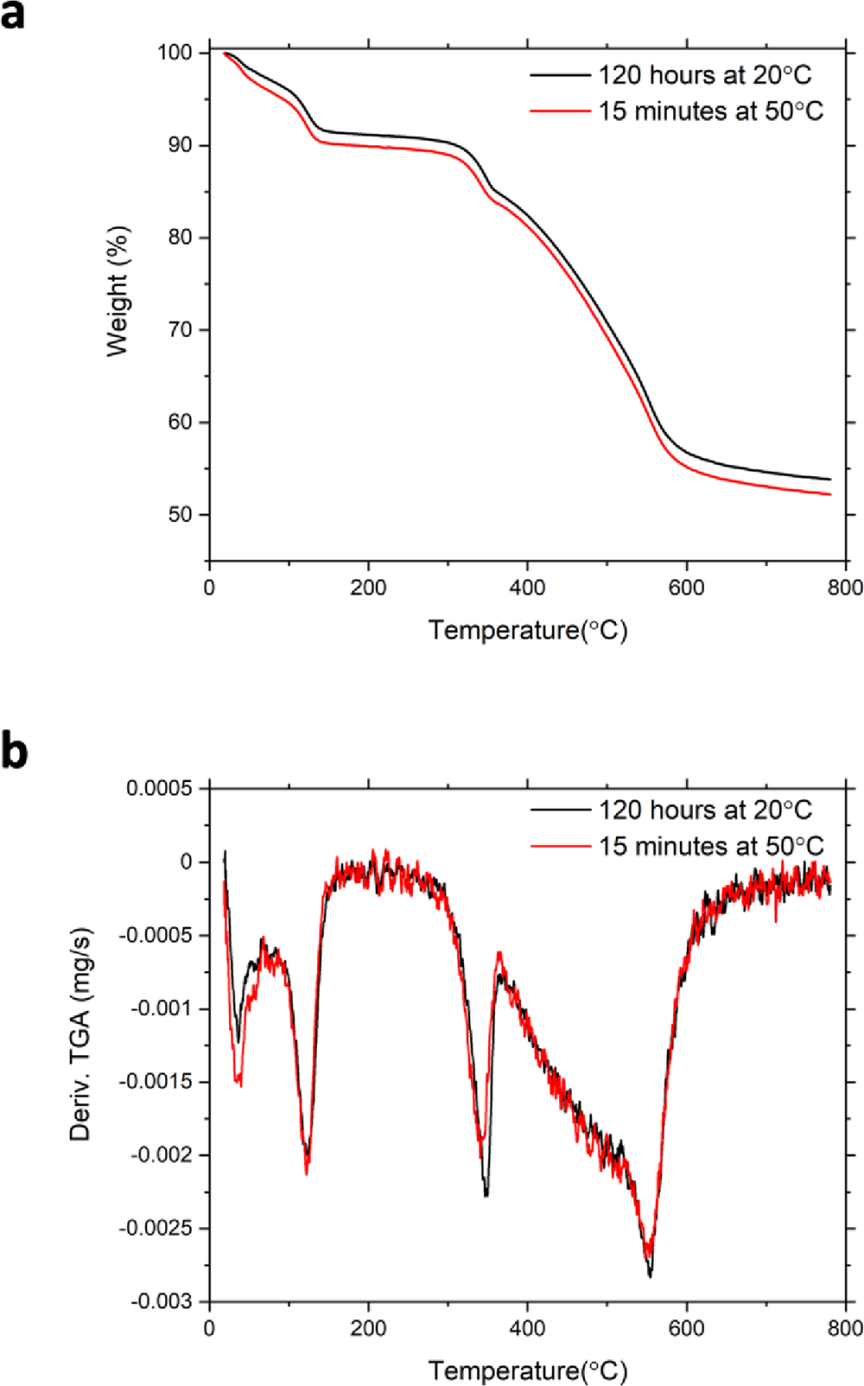
(a) Weight loss and (b) derivative resulting from the thermogravimetric analysis of θ-ZrP intercalated with Rhodamine 6G at room temperature and 50 °C.

The Rhodamine 6G content of each sample is reported in Tables S2 and S3. For the room temperature intercalation, the uptake of Rhodamine 6G systematically increases until reaching a maximum uptake of 0.35 mol after 5 days of mixing. These findings agree well with the diffraction patterns that transition from a mixed phase to a single-phase material and from less to more ordered with time. However, the intercalation compounds produced from the reaction at 50 °C all display Rhodamine 6G contents superior to the room temperature maximum. In fact, an uptake of 0.37 mol was achieved in 15 min at elevated temperature, representing a 5.7% increase in loading in only 0.2% of the time required at room temperature. At 15 min the room temperature intercalation yields an uptake of only 0.21 mol of Rhodamine 6G, therefore the elevated temperature intercalation shows a 76% increase in uptake at the 15 min time scale. These findings coincide with the results from HT ZrP where the continuous addition method at elevated temperature often led to increased ion uptake at much shorter time scales relative to the equilibrium-based approach. It appears then that the insights gained from the study of HT ZrP can be applied to different phases of ZrP, diverse intercalation mechanisms, and likely other layered solids. We recommend researchers explore the use of elevated temperature to optimize intercalation and ion-exchange reactions.

## Conclusion

This work presents the first systematic investigation of the ion-exchange behavior of HT ZrP, establishing clear relationships between synthesis-dependent physicochemical properties, reaction conditions, and ion-exchange behavior. The CEC was strongly influenced by both titration method and structural order, with less crystalline preparations and the incremental addition method achieving values closest to the theoretical capacity of α-ZrP. Elevated reaction temperatures further improved the CEC, particularly for crystalline samples using the continuous addition method, highlighting the role of kinetic and transport limitations imposed by the structure. These findings demonstrate that ion exchange is not governed solely by bulk crystallinity, but by the interplay of structural order, particle morphology, thermodynamics, and mass transport.

The practical utility of these insights was demonstrated through the rapid intercalation of Rhodamine 6G into θ-ZrP. At 50 °C superior uptake of the guest molecule was observed over the entire course of the intercalation; notably, an uptake of 0.37 mol of Rhodamine 6G was obtained in only 15 min, surpassing the uptake achieved after 120 h at room temperature. Overall, this work correlates the ion exchange behavior of α-ZrP to its synthesis-dependent structural and physicochemical properties, providing a predictive framework for the rational design of materials with distinctive exchange behavior or the optimization of processes such as intercalation in existing materials.

## Supplementary Material



## Notes

TGA of α-ZrP prepared by hydrothermal synthesis, van’t Hoff plots for the ion exchange of each synthesized material, and TGA data from the intercalation of Rhodamine 6G into θ-ZrP (PDF)

## †

University of Central Florida, Department of Chemistry, Orlando, Florida 32816, United States

## ‡

Boston University, Graduate Medical Sciences, 72 East Concord Street, Boston, Massachusetts 02118, United States.

## §

Meharry Medical College, School of Dentistry, 1005 Dr. DB Todd Jr Blvd, Nashville, Tennessee 37203, United States.

## Abbreviations

fwhm: full width at half-maximum
α-ZrP: alpha zirconium phosphate
HT ZrP: hydrothermal alpha zirconium phosphate or alpha zirconium phosphate prepared by hydrothermal synthesis
PXRD: powder X-ray diffraction
TGA: thermogravimetric analysis
CEC: cation exchange capacity
