# Supplementary material for: Ion Exchange of α‑Zirconium Phosphate Prepared by Hydrothermal Synthesis
Source: ACS Omega. 2026 Mar 19;11(12):19134–45. doi: 10.1021/acsomega.5c11825 (PMC13044664; doi:10.1021/acsomega.5c11825)
Supplement: Supplementary file 1 [file ao5c11825_si_001.pdf]

Ion Exchange of α-Zirconium Phosphate Prepared by Hydrothermal Synthesis

*Cecilia Hernandez, John L. Reynoso, Christian A. Santiago, Asia Sinclair, Sophia S. Bolan, and Brian M. Mosby**

Department of Chemistry, Rollins College, United States, 1000 Holt Ave., Winter Park, Florida 32789

Email: bmosby@rollins.edu

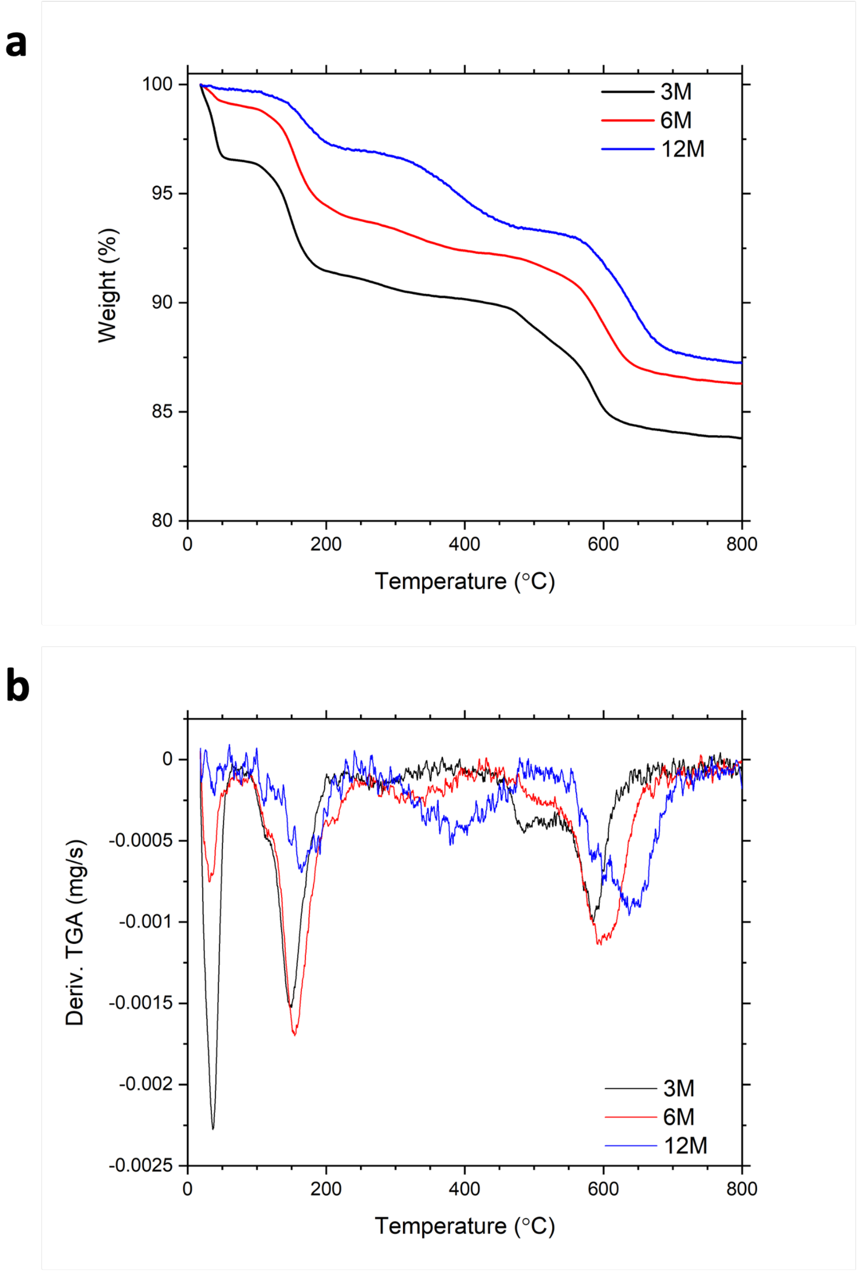

Figure S1. The (a) weight loss and (b) derivatives resulting from the thermogravimetric analysis of HT ZrP.

Table S1. The cation exchange capacity of 12 M HT ZrP determined from the inflection point corresponding to the neutralization of the second proton in the first derivative of the titration curve.

	Cation Exchange Capacity (meq OH^{-}/g ZrP)
Experiment 1	6.177
Experiment 2	6.141
Experiment 3	6.104
Average	6.140
Standard Deviation	0.036

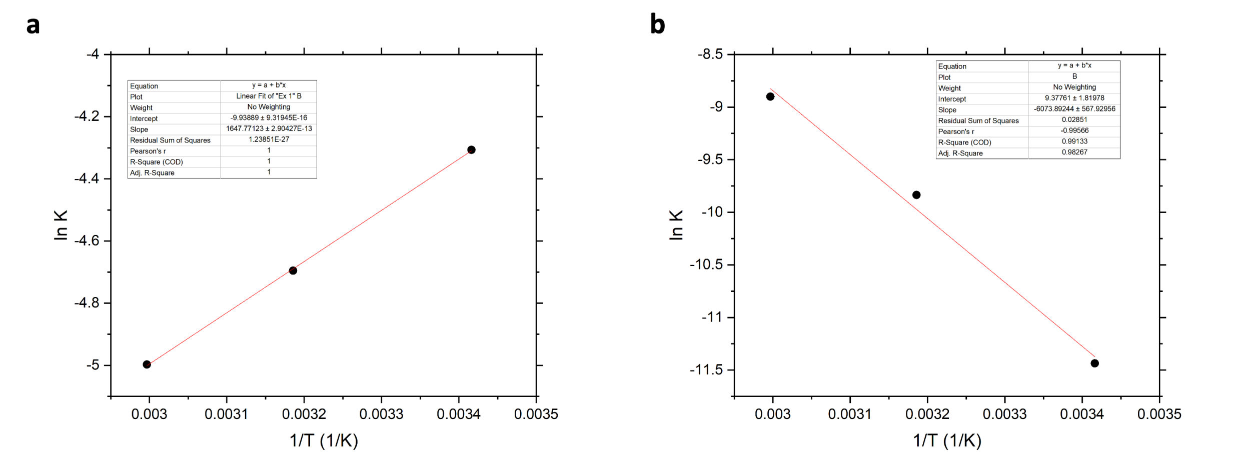

Figure S2. Van't Hoff plots for the exchange of the (a) first and (b) second proton during the titration of 3 M HT ZrP with sodium hydroxide.

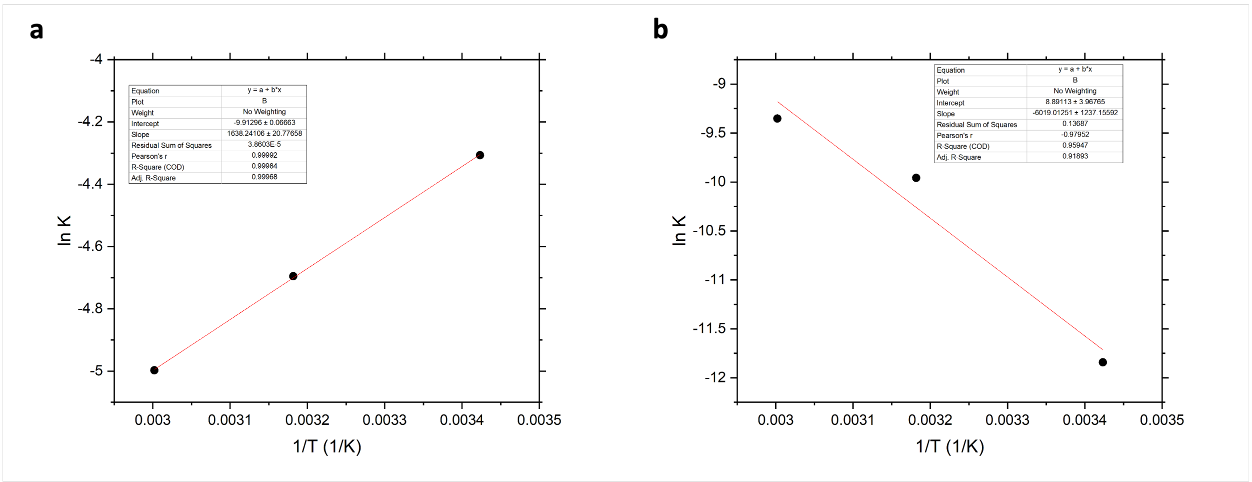

Figure S3. Van’t Hoff plots for the exchange of the (a) first and (b) second proton during the titration of 6 M HT ZrP with sodium hydroxide.

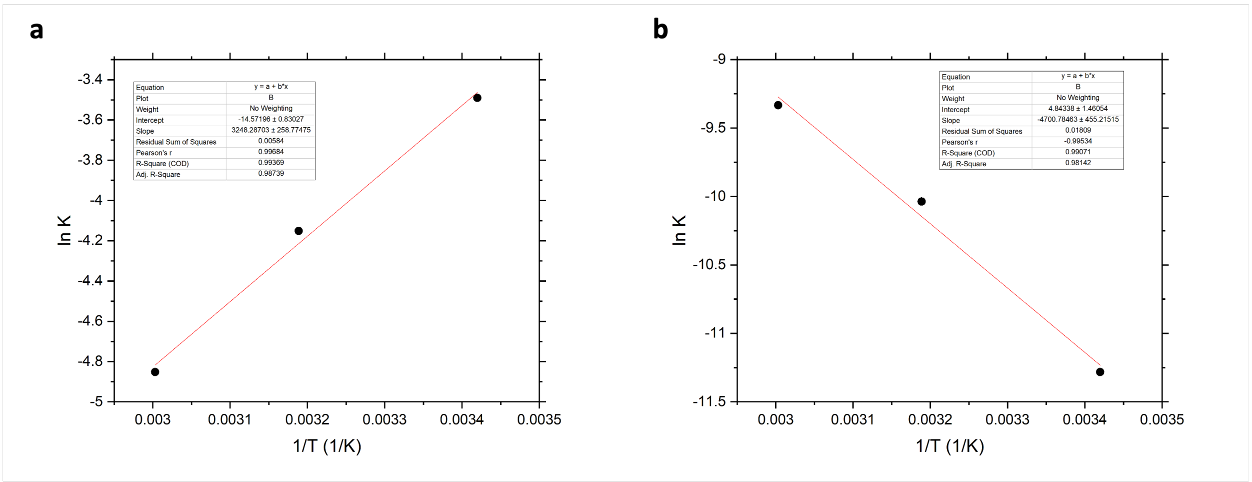

Figure S4. Van’t Hoff plots for the exchange of the (a) first and (b) second proton during the titration of 12 M HT ZrP with sodium hydroxide.

Table S2. Total weight loss and moles of Rhodamine 6G determined by thermogravimetric analysis of the solids produced from intercalation of Rhodamine 6G into θ-ZrP at room temperature.

Reaction Time (hours)	Weight Loss (%)	Mol Rhodamine 6G per mole of Zr
0.25	36.94	0.21
0.50	41.57	0.27
1	42.41	0.28
24	45.82	0.34
72	46.03	0.35
120	46.33	0.35

Table S3. Total weight loss and moles of Rhodamine 6G determined by thermogravimetric analysis of the solids produced from intercalation of Rhodamine 6G into θ-ZrP at 50°C.

Reaction Time (hours)	Weight Loss (%)	Mol Rhodamine 6G per mole of Zr
0.25	48.19	0.37
0.50	46.77	0.36
1	46.61	0.36
24	47.13	0.37
72	48.44	0.39
120	48.03	0.39
